# Anatomical roadmap of the thenar motor branches: key insights for distal nerve transfers

**DOI:** 10.1177/17531934251389494

**Published:** 2025-10-24

**Authors:** Esmee Kwee, Caroline A Hundepool, Dominic M Power, Liron S Duraku, J Michiel Zuidam

**Affiliations:** 1 Department of Plastic, Reconstructive Surgery and Hand Surgery, Erasmus Medical Centre, Rotterdam, The Netherlands; 2 Department of Hand and Peripheral Nerve Surgery, Queen Elizabeth Hospital Birmingham, UK; 3 Department of Plastic, Reconstructive Surgery and Hand Surgery, Amsterdam University Medical Centre, Amsterdam, The Netherlands

**Keywords:** Distal nerve transfers, median nerve, opponens pollicis, peripheral nerve injury, thenar motor branches, ulnar nerve

## Abstract

Ultra-distal nerve transfers require precise anatomical knowledge. The thenar motor branches were mapped in 26 cadaveric hands. Three anatomical variations were found. The opponens pollicis branch showed consistent anatomy and optimal donor characteristics for transfer for treatment of ulnar nerve palsy.

Ultra-distal nerve transfers have emerged as a valuable option to restore function after ulnar or median nerve injuries (Bertelli, 2024; Bertelli et al., 2018). Their success, however, depends on precise anatomical knowledge. Surgeons performing ultra-distal nerve transfers need to know exactly where the motor branches in the hand lie, what anatomical variations to expect, and which branches are expendable. The thenar motor nerves are especially important, frequently serving as both donors and recipients during reconstruction.

In this study, 26 human cadaveric hands were dissected. Following carpal tunnel release, the median nerve was exposed and traced distally to identify its thenar motor branches: abductor pollicis brevis (APB), opponens pollicis (OP) and flexor pollicis brevis (FPB). Each branch was carefully dissected to its motor entry point. The length and diameter of each branch were measured from the distal border of the flexor retinaculum to its motor entry point using a digital caliper, and all measurements were verified by two authors.

Three branching patterns were identified (Figure 1). Type 1 consisted of a main thenar trunk supplying FPB, APB and OP (12/26). Type 2 involved a main thenar trunk supplying APB and OP, with FPB supplied by a separate more distal branch (10/26). Type 3 involved no trunk, with all branches arising independently (4/26). The mean measurements for each branch across patterns are summarized in Table 1.

**Figure 1.**
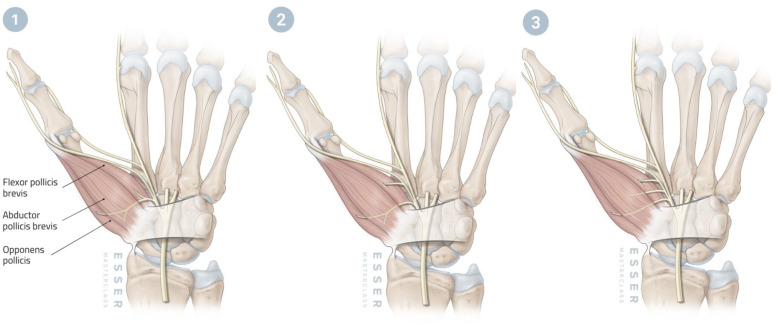
Illustrations of the three types of distribution patterns of the thenar motor branches of the median nerve.

**Table 1.** Mean lengths (mm) (SD) and mean diameters (mm) (SD) per branching patterns of the thenar motor branches.

Branch	Type 1	Type 2	Type 3
APB	11 (4) 0.9 (0.3)	13 (4) 0.8 (0.4)	18 (6) 0.9 (0.1)
OP	13 (4) 1.0 (0.3)	16 (5) 1.0 (0.4)	19 (4) 0.9 (0.2)
FPB trunk	13 (6) 0.8 (0.2)		
FPB distal	*18 (5) 0.7 (0.3)	14 (5) 0.7 (0.2)	18 (5) 0.9 (0.2)

* In two cases, an additional distal FPB branch supplied the FPB;

APB: abductor pollicis brevis; OP: opponens pollicis; FPB: flexor pollicis brevis; SD: standard deviation.

Donor suitability was defined by two factors: anatomical consistency and functional expendability. The APB and OP branches were the most consistent, usually arising together from a main trunk. The FPB branch was highly variable: sometimes from the trunk, often from a distal branch, and occasionally dual or absent, making it unreliable. Between the APB and OP branches, only the OP can be considered expendable. The APB is the key muscle for thumb opposition and must be preserved (Littler, 1949). The OP contributes to but does not independently generate opposition. Sacrificing the OP branch is therefore unlikely to compromise thumb function, while providing a consistent donor.

Having established its suitability, the next question is how to find the OP branch intraoperatively? In 22 of 26 hands, the OP branch arose from the main thenar trunk. This trunk originated consistently from the antero-radial side of the median nerve, branching at the distal border of the flexor retinaculum. No trans-retinacular course was seen. The OP branch lay in a deeper plane than the APB branch; gentle elevation of APB muscle was required to reveal its course. The mean length of this OP branch to its motor entry point was 14mm (SD 4.5), with a diameter of 1.0mm (SD 0.3). In the remaining four hands, the OP branch originated directly from the median nerve as a solitary branch. It was the first thenar branch to arise, before APB and FPB. In this pattern, the OP measured 19mm (SD 4.4), with a diameter of 0.9mm (SD 0.2).

Finally, the feasibility of ultra-distal transfers was assessed in six dissections. The OP branch consistently reached the deep terminal motor branch of the ulnar nerve without tension, supporting its use to restore pinch after ulnar nerve injuries (Figure S1). Likewise, the thenar branches served as reliable recipients for restoring thumb opposition in median injuries through tension-free coaptation of the abductor digiti minimi branch (Figure S2).

Previous anatomical studies have focused on describing anatomical variations, representing two or three variants, but lacked detailed measurements or evaluation within the context of nerve transfers (Olave et al., 1995). As ultra-distal nerve transfers in the hand become more prevalent, the anatomical insights presented in this study offer surgeons clear guidance for intraoperative decision-making and reliable identification of the OP branch as the optimal donor for transfers, and ultimately improve clinical outcomes.

## Supplemental Material

sj-docx-1-jhs-10.1177_17531934251389494 – Supplemental material for Anatomical roadmap of the thenar motor branches: key insights for distal nerve transfers Supplemental material, sj-docx-1-jhs-10.1177_17531934251389494 for Anatomical roadmap of the thenar motor branches: key insights for distal nerve transfers by Esmee Kwee, Caroline A Hundepool, Dominic M Power, Liron S Duraku and J Michiel Zuidam in Journal of Hand Surgery (European Volume)

sj-docx-2-jhs-10.1177_17531934251389494 – Supplemental material for Anatomical roadmap of the thenar motor branches: key insights for distal nerve transfers Supplemental material, sj-docx-2-jhs-10.1177_17531934251389494 for Anatomical roadmap of the thenar motor branches: key insights for distal nerve transfers by Esmee Kwee, Caroline A Hundepool, Dominic M Power, Liron S Duraku and J Michiel Zuidam in Journal of Hand Surgery (European Volume)
