# Supplementary material for: Anatomical roadmap of the thenar motor branches: key insights for distal nerve transfers
Source: J Hand Surg Eur Vol. 2025 Oct 24;51(6):820–2. doi: 10.1177/17531934251389494 (PMC13216568; doi:10.1177/17531934251389494)
Supplement: sj-docx-1-jhs-10.1177_17531934251389494 – Supplemental material for Anatomical roadmap of the thenar motor branches: key insights for distal nerve transfers [file sj-docx-1-jhs-10.1177_17531934251389494.docx]

**Figure S1.** Illustrations of the opponens (OP) motor branch end-to-end transfer to the deep terminal division of the ulnar nerve (DTDUN) for restoring pinch after ulnar nerve injuries.


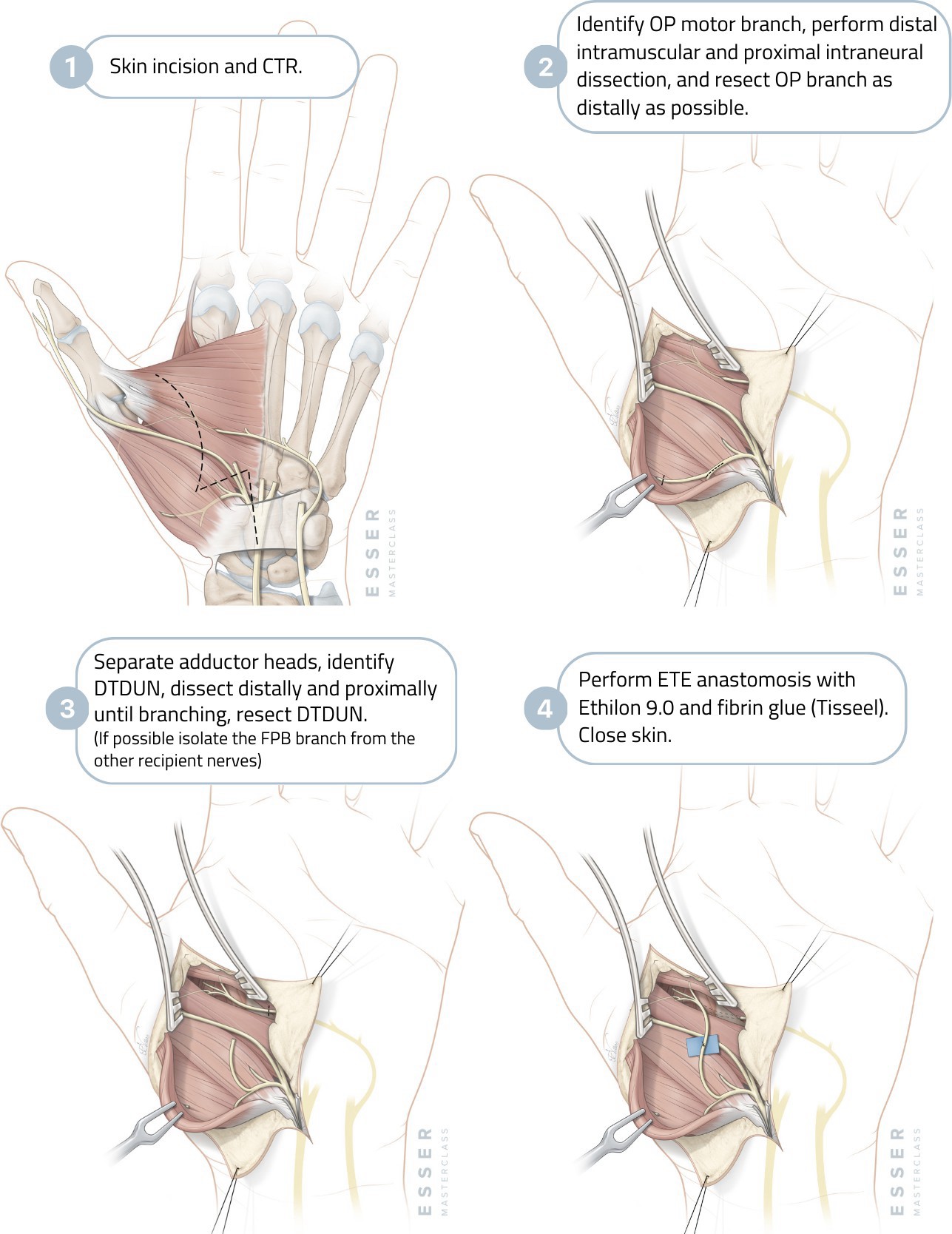


CTR, Carpal tunnel release; OP, opponens pollicis; DTDUN, deep terminal division of the ulnar nerve; FPB, flexor pollicis brevis; ETE, end-to-end.
