# Supplementary material for: Anatomical roadmap of the thenar motor branches: key insights for distal nerve transfers
Source: J Hand Surg Eur Vol. 2025 Oct 24;51(6):820–2. doi: 10.1177/17531934251389494 (PMC13216568; doi:10.1177/17531934251389494)
Supplement: sj-docx-2-jhs-10.1177_17531934251389494 – Supplemental material for Anatomical roadmap of the thenar motor branches: key insights for distal nerve transfers [file sj-docx-2-jhs-10.1177_17531934251389494.docx]

**Figure S2.** Illustrations of the abductor digiti minimi (ADM) motor branch end-to-end transfer to the main thenar nerve for restoring thumb opposition after median nerve injury.


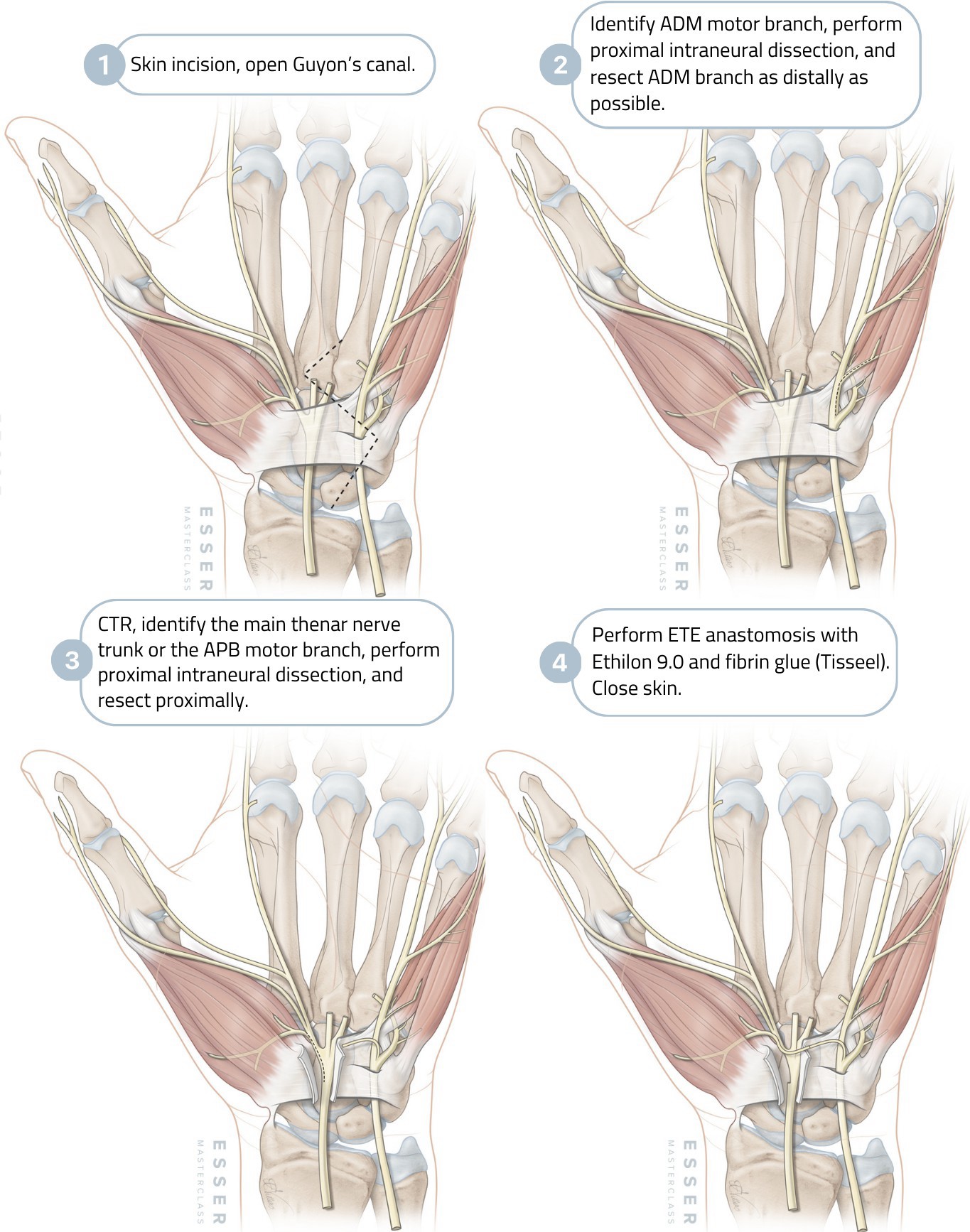


ADM, Abductor digiti minimi; CTR, Carpal tunnel release; APB, Abductor pollicis brevis; ETE, end-to-end.
